# Phaseolorin J Alleviates Cellular Inflammation and Oxidative Stress by Inhibiting NLRP3 Inflammasome Expression via the Nrf2/HO-1 Pathway

**DOI:** 10.3390/md24040130

**Published:** 2026-03-31

**Authors:** Yuanjie Chen, Ting Feng, Xiaojing Li, Jing Xu, Juren Cen

**Affiliations:** 1 Hainan Province Key Laboratory of One Health, Collaborative Innovation Center of Life and Health, School of Life and Health Sciences, Hainan University, Haikou 570228, China; 2 Collaborative Innovation Center of Ecological Civilization, School of Chemistry and Chemical Engineering, Hainan University, Haikou 570228, China

**Keywords:** phaseolorin J, mangrove endophytic fungi, anti-inflammatory activity, Nrf2/HO-1, NLRP3, oxidative stress, RAW264.7

## Abstract

Phaseolorin J (TT-55), a chromone compound isolated and purified from the fermentation products of Phomopsis asparagi DHS-48, is an endophytic fungus obtained from mangrove forests. Preliminary experimental studies have revealed its potent antioxidant and anti-inflammatory activities, though its mechanism of action remains unclear. In this study, we aimed to investigate the molecular mechanisms underlying the antioxidant and anti-inflammatory effects of TT-55, following initial evidence of its potency, by employing an LPS-induced RAW264.7 macrophage model in vitro. The results revealed that in the LPS-induced inflammatory model of RAW264.7 cells, the TT-55 dose dependently inhibited the expression of LPS-induced inflammatory cytokines (TNF-α, IL-18, IL-1β, IL-6) and the production of oxidative stress markers (reactive oxygen species, SOD, MDA). Following combined treatment with the Nrf2 pathway inhibitor ML385 and TT-55, the inhibitory effects of TT-55 on inflammatory cytokines and oxidative stress markers were reversed by ML385. Meanwhile, ML385 also attenuated the ability of TT-55 to suppress LPS-induced upregulation of NLRP3 inflammasome-related genes. In conclusion, TT-55 may exert its antioxidant and anti-inflammatory effects by activating the Nrf2/HO-1 signaling pathway and suppressing the upregulation of NLRP3 inflammasome-related genes.

## 1. Introduction

Inflammation is recognized as one of the primary protective responses of the innate immune system, arising from activation processes within the mammalian immune system. It is closely related to the body’s defense mechanisms that respond to chemical, biological, or physical infections and injuries [1,2]. Inflammation serves as a key driver in the progression of various chronic diseases/conditions, including diabetes, cancer, cardiovascular disease, eye diseases, arthritis, obesity, autoimmune disorders, and inflammatory bowel disease [3]. Uncontrolled inflammatory responses may evolve into persistent issues that significantly contribute to the development of various diseases, including asthma, diabetes, Alzheimer’s disease, atherosclerosis, rheumatoid arthritis, and even certain cancers. Therefore, the control of chronic inflammation and maintenance of a balance between inflammatory and anti-inflammatory processes within the body is crucial [4,5,6]. Currently, steroidal and non-steroidal anti-inflammatory drugs (NSAIDs)—which exert anti-inflammatory, analgesic, and other therapeutic effects remain the most commonly used classic treatments for inflammation in clinical practice. However, long-term use of these drugs may cause various adverse reactions, such as gastrointestinal damage (gastric ulcers, bleeding, etc.), hepatic and renal dysfunction, and dermatological disorders [7,8], which preclude their long-term use. In recent years, natural products and their derived small-molecule compounds have garnered extensive attention as promising drug sources due to their unique chemical structures, low toxicity, and multi-target regulatory capabilities, rendering them invaluable in drug discovery for inflammation treatment [9]. However, identifying nontoxic natural products with superior efficacy to replace traditional anti-inflammatory drugs remains an unmet challenge in the clinical management of inflammation-related diseases. Nuclear Factor Erythroid 2-Related Factor 2 (Nrf2) serves as the primary transcriptional regulator for numerous genes involved in oxidative stress adaptation responses. Endogenous free radicals and reactive oxygen species (ROS) can directly or indirectly damage cellular components such as proteins, lipids, and DNA. To counteract these detrimental effects, the body has evolved a complex oxidative stress response system to mitigate cellular damage [10,11]. Furthermore, the Nrf2/HO-1 signaling pathway has emerged as a critical therapeutic target in cancer, neurodegenerative diseases, and numerous autoimmune and inflammatory disorders [12,13]. However, ROS induces conformational changes in Keap1 by modifying a key cysteine residue within its structure, thereby preventing effective Keap1-Nrf2 binding and inhibiting Keap1’s role in promoting Nrf2 degradation [14]. Consequently, Nrf2 accumulates in the cytoplasm and subsequently translocates to the nucleus. There, it binds to Maf proteins and associates with AREs present in the upstream promoter regions of numerous antioxidant genes [15]. The Nrf2-ARE complex acts as a transcription activator, recruiting other coactivators of the transcriptional machinery, such as RNA polymerase II, to the promoter regions of these genes [16,17]. Nrf2 binding to AREs leads to the transcriptional activation of multiple genes encoding proteins with antioxidant, anti-inflammatory, and cytoprotective functions [18]. ROS were also implicated in the mechanisms governing the assembly and activation of the NLRP3 inflammasome, thereby contributing to tissue inflammation and the elicitation of immune responses [19,20]. The NLRP3 inflammasome was composed of three core constituents: NLRP3 protein, caspase-1, and apoptosis-associated speck-like protein containing a CARD (ASC). The structural architecture of this complex endowed it with a pivotal role in inflammatory responses [21]. Upon activation by ROS, NLRP3 recruited ASC and activated caspase-1, which in turn promoted the maturation and secretion of pro-inflammatory cytokines, including interleukin-1β (IL-1β) and interleukin-18 (IL-18), and participated in diverse inflammatory processes in vivo [22]. As a critical regulator in the oxidative stress signaling cascade, Nrf2 established a molecular linkage by mediating the interplay between ROS and NLRP3 inflammasome activation, which had emerged as a prominent focus in investigations of inflammatory mechanisms [23]. Three small-molecule inhibitors targeting this pathway have been developed and approved for marketing, such as monomethyl fumarate, diroximel fumarate, and omevaloxolone; however, all of these are chemically synthesized drugs, and to date, no natural product-derived drugs targeting Nrf2 have been approved [24,25,26].

Natural products are a rich and valuable source of bioactive molecules with diverse pharmacological activities and have long served as important lead compounds in the development of anti-inflammatory and antioxidant agents. Compared with synthetic compounds, natural products typically exhibit good biocompatibility and multi-target regulatory properties, making them highly attractive for the treatment of inflammation-related diseases [27]. Mangroves constitute complex and vital marine wetland ecosystems, predominantly distributed in tropical and subtropical intertidal estuarine zones. They nurture affluent microbial communities including fungi, actinomycetes, bacteria, cyanobacteria, algae, and protozoa. Endophytic fungi from mangrove ecosystems represent one of the most promising sources of bioactive natural products [28]. Their distinctive structural frameworks and secondary metabolites have made them a focal point for active natural compounds [29]. Among these, the mangrove endophytic strain *Phomopsis asparagi* DHS-48, isolated from fresh roots of Rhizophora mangle, has drawn our attention. We have identified a series of immunomodulatory compounds from DHS-48, including xanthones, cytochalasins, and furanone derivatives [30,31,32], alongside alkaloids, sterols, and other types of polyketides exhibiting acetylcholinesterase (AChE) inhibitory activity [33]. To uncover additional unknown secondary metabolites, epigenetic manipulation was performed on this fungus using the histone deacetylase (HDAC) inhibitor sodium butyrate and the DNA methyltransferase (DNMT) inhibitor 5-aza-cytidine (5-aza) to activate concealed or silenced genes. From this process, phaseolorin J (TT-55) was isolated and extracted. TT-55 is a natural product extracted and isolated from the secondary metabolites of endophytic fungi in mangroves. It possesses a unique structural scaffold distinct from many known anti-inflammatory compounds. Despite its promising structural features, the biological activity and underlying mechanism of TT-55 in regulating inflammation and oxidative stress remain largely unexplored. This compound exhibited moderate inhibitory effects on the proliferation of T lymphocytes induced by concanavalin A (ConA) and on mouse B lymphocytes stimulated by lipopolysaccharide (LPS) [34]. Preliminary screening of TT-55 for anti-inflammatory activity revealed its capacity to suppress LPS-induced nitric oxide (NO) production and downregulate the expression of the inflammatory cytokine IL-1β. In this study, we aimed to explore the potential mechanism by which TT-55 regulates the expression of inflammatory mediators and oxidative stress factors in LPS-stimulated RAW 264.7 macrophages through a signaling cascade involving the Nrf2/HO-1 pathway. MCC950 is a well-characterized, selective NLRP3 inflammasome inhibitor that also exhibits significant anti-inflammatory and antioxidant activity [35]. In this experiment, we selected it as a positive control; its primary function is to validate the validity of this experimental system and to observe the differences in anti-inflammatory and antioxidant activity between TT-55 and targeted inhibitors such as the NLRP3 inhibitor. This will assist our subsequent research into NLRP3-targeted inhibitory activity, ensuring the reliability and credibility of the experimental results.

## 2. Results

### 2.1. TT-55 Inhibits LPS-Induced Inflammatory Cytokine Expression in RAW264.7 Cells

**Figure 1:**
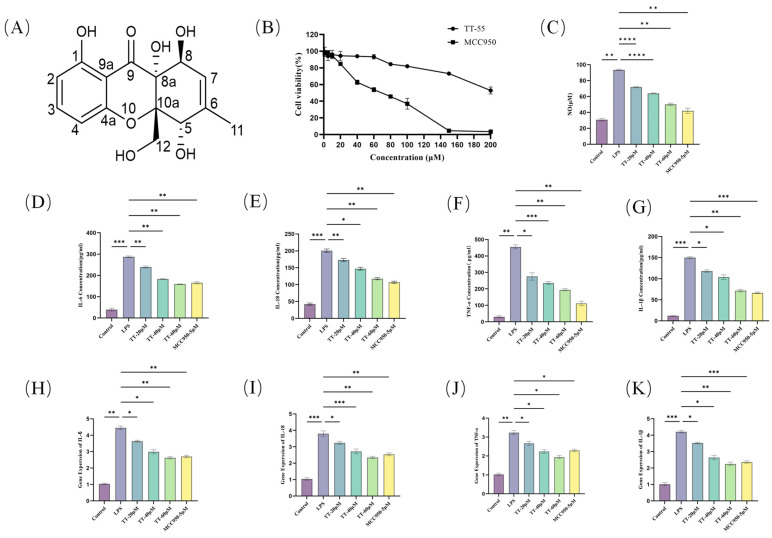
TT-55 inhibits LPS-induced inflammation in RAW264.7 cells. (**A**) Chemical structure of TT-55. (**B**) Cytotoxic effects of TT-55 and MCC950 on RAW264.7 cells. (**C**) TT-55 inhibits NO production in RAW264.7 cells. (**D**–**G**) TT-55 suppresses LPS-induced inflammatory cytokine expression (IL-6, IL-18, IL-10, TNF-α, and IL-1β) in RAW264.7 cells. (**H**–**K**) TT-55 inhibits mRNA expression of LPS-induced inflammatory cytokines IL-6, IL-18, TNF-α, and IL-1β in RAW264.7 cells. Each experiment was independently performed in triplicate at minimum, and all results are presented as mean ± standard deviation. * *p* < 0.05, ** *p* < 0.01, *** *p* < 0.001, and **** *p* < 0.0001 compared to the LPS group.

To determine the safe concentration range of TT-55 for RAW264.7 cells, the CCK-8 assay was employed to assess its cytotoxic effects. RAW264.7 cells were incubated with various concentrations of TT-55 and MCC950 for 24 h. Figure 1B illustrates the toxic effects of TT-55 and MCC950 on RAW264.7 cells. TT-55 was well tolerated by RAW264.7 cells, showing negligible cytotoxic effects at concentrations below 60 μM. Cell viability began to decrease at 80 μM, with a half maximal inhibitory concentration (IC_50_) of 194.7 μM. Therefore, we selected concentrations with negligible cellular toxicity (20 μM, 40 μM, 60 μM) for subsequent experiments to avoid drug-induced cellular toxicity. In contrast, at a concentration of 60 μM, MCC950 reduced the cell survival rate to only 53.8%, while at 150 μM, almost all cells died. NO release assays revealed TT-55’s effects on LPS-induced inflammatory responses in RAW264.7 cells. Figure 1C showed that LPS stimulation induced substantial NO release, with TT-55 concentrations exhibiting a clear dose–response relationship. NO production progressively decreased with increasing compound concentration. The IC_50_ for TT-55’s NO inhibition was 43.15 μM. The enzyme-linked immunosorbent assay (ELISA) was used to detect changes in cellular inflammatory factor expression, with the results depicted in Figure 1D–G. Compared to the blank group, the LPS group exhibited substantial release of several inflammatory factors. Following TT-55 addition, the expression levels of various inflammatory factors in the cell culture supernatant were downregulated in a dose-dependent manner. qRT-PCR was adopted to measure mRNA expression of IL-1β, IL-6, IL-18, and TNF-α. TT-55 significantly reduced LPS-induced pro-inflammatory cytokine mRNA expression in RAW264.7 cells. This inhibitory effect was dose-dependent, as illustrated in Figure 1H–K. Taking the above results together, it can be seen that TT-55 has a relatively weaker inhibitory effect on inflammatory cytokines and NO production than MCC950; however, in RAW264.7 cells, TT-55 exhibits significantly lower cytotoxicity, suggesting that it has a better safety profile than MCC950.

### 2.2. TT-55 Inhibits LPS-Induced Oxidative Stress in RAW264.7 Cells

**Figure 2:**
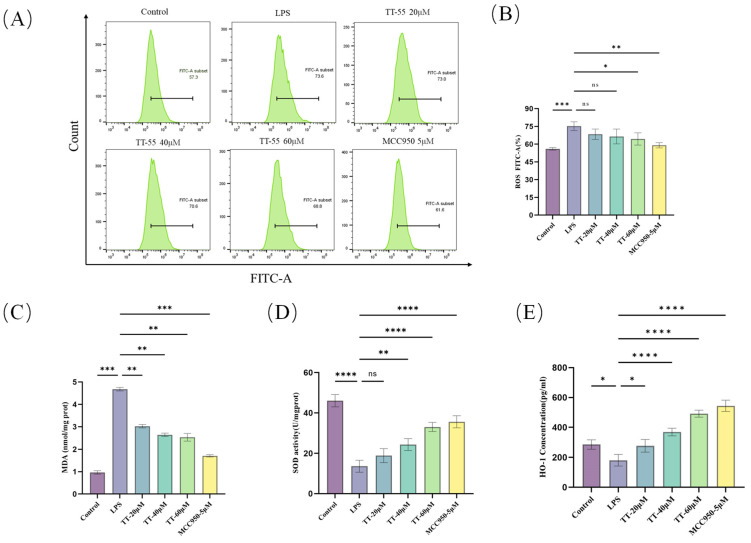
TT-55 pretreatment alleviates LPS-induced oxidative stress in RAW264.7 cells. (**A**,**B**) Flow cytometric analysis of TT-55 effects on LPS-induced ROS in RAW264.7 cells. (**C**) TT-55 effects on LPS-induced MDA in RAW264.7 cells. (**D**) TT-55 effects on LPS-induced SOD in RAW264.7 cells. (**E**) TT-55 modulates LPS-induced HO-1 expression in RAW264.7 cells. Each experiment was independently performed in triplicate at minimum, and all results are presented as mean ± standard deviation. * *p* < 0.05, ** *p* < 0.01, *** *p* < 0.001, and **** *p* < 0.0001 compared to the LPS group.

Flow cytometry analysis showed that the TT-55 dose-dependently attenuated intracellular ROS levels in LPS-induced RAW 264.7 cells compared to the LPS-treated group (Figure 2A,B). Specifically, ROS levels were dose-dependently decreased from 73.6% (LPS group) to 73% (LPS + 20 μM TT-55), 70.6% (LPS + 40 μM TT-55), and 68.8% (LPS + 60 μM TT-55). This occurred in the context of an LPS-induced oxidative stress state characterized by elevated MDA and suppressed SOD and HO-1 activities. As shown in Figure 2C–E, compared to the LPS group, TT-55 treatment significantly enhanced SOD and HO-1 activity while markedly reducing MDA activity, exhibiting a dose-dependent effect. These results indicated that TT-55 inhibited the activation of oxidative stress factors in LPS-induced RAW264.7 cells, reducing ROS and MDA production and enhancing SOD and HO-1 expression. Therefore, TT-55 exhibited significant antioxidant properties and exerted a protective effect against LPS-induced oxidative stress in RAW264.7 cells.

### 2.3. TT-55 Upregulates Nrf2/HO-1 Activation Levels

**Figure 3:**
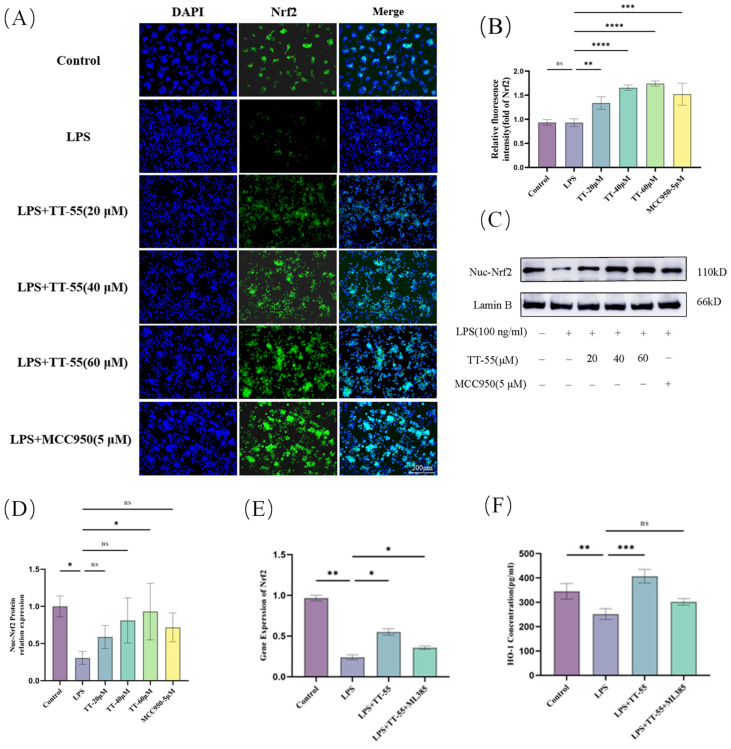
Effects of TT-55 treatment on the Nrf2/HO-1 pathway. (**A**,**B**) Immunofluorescence analysis of Nrf2 nuclear translocation. (**C**,**D**) Western blot analysis of Nrf2 protein levels. (**E**) qPCR analysis of Nrf2 mRNA expression in RAW 264.7 macrophages under Nrf2 pathway inhibitor treatment. (**F**) Analysis of HO-1 expression levels in RAW 264.7 macrophages treated with TT-55 under Nrf2 pathway inhibitor conditions. Each experiment was independently performed in triplicate at minimum, and all results are presented as mean ± standard deviation. * *p* < 0.05, ** *p* < 0.01, and *** *p* < 0.001, and **** *p* < 0.0001compared with the LPS group.

The image captured by the inverted fluorescence microscope is shown in Figure 3A,B. Compared to the blank group, LPS stimulation reduced Nrf2 nuclear translocation and inhibited Nrf2 atomic entry. However, compared to the LPS group, all TT-55-treated groups (20, 40, and 60 μM) significantly enhanced Nrf2 nuclear translocation. Meanwhile, intense green fluorescence observed in these groups further confirmed this effect. Concurrent Western blot analysis of Nrf2 protein levels and qRT-PCR analysis of Nrf2 gene expression confirmed these immunofluorescence findings, demonstrating that TT-55 treatment increased Nrf2 expression levels in RAW264.7 cells (Figure 3C–E). As shown in Figure 3F, TT-55 treatment enhanced HO-1 expression levels, consistent with Nrf2, and the enhancing effect of TT-55 was suppressed by ML385. Therefore, TT-55 may activate the Nrf2/HO-1 signaling pathway by promoting Nrf2 nuclear translocation.

### 2.4. TT-55 Inhibits LPS Induced Inflammatory Mediator and ROS Expression in RAW264.7 Cells via the Nrf2/HO-1 Pathway

**Figure 4:**
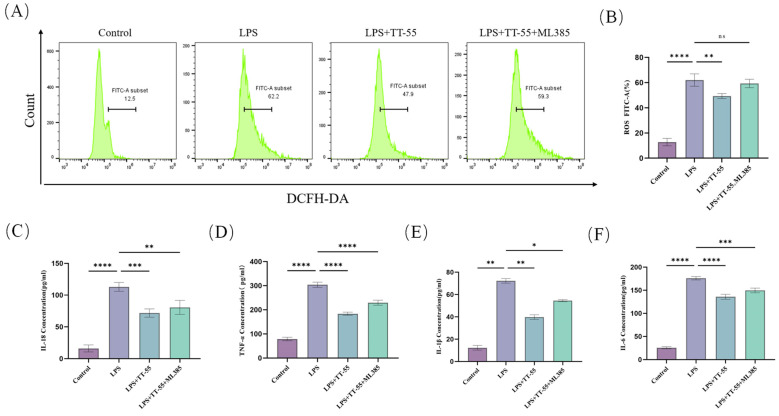
TT-55 upregulates the Nrf2/HO-1 signaling pathway to inhibit LPS-induced inflammatory cytokine and ROS expression in RAW264.7 cells. (**A**,**B**) Flow cytometry analysis of TT-55’s effect on LPS-induced ROS in RAW264.7 cells under Nrf2 pathway inhibitor treatment. (**C**–**F**) TT-55 modulates LPS-induced inflammatory cytokines (IL-18, TNF-α, IL-1β, IL-6) in RAW264.7 cells under Nrf2 pathway inhibitor treatment, as assessed by ELISA. Each experiment was independently performed in triplicate at minimum, and all results are presented as mean ± standard deviation. * *p* < 0.05, ** *p* < 0.01, *** *p* < 0.001, and **** *p* < 0.0001 compared with the LPS group.

Following co-treatment of RAW264.7 cells with TT-55 and the Nrf2 pathway inhibitor ML385, ROS expression was detected via flow cytometry, as shown in Figure 4A,B. LPS stimulation induced substantial ROS release in RAW264.7 cells. TT-55 treatment significantly reduced cellular ROS levels compared to the LPS-treated group, decreasing from 62.2% to 47.9%. However, co-addition of ML385 markedly attenuated TT-55’s inhibitory effect, leading to a significant increase in ROS expression. These results indicate that TT-55 suppresses LPS-induced activation of oxidative stress factors in RAW264.7 cells by activating the Nrf2 pathway, thereby reducing ROS production.

ELISA was used to assess the effect of TT-55 on LPS-induced pro-inflammatory cytokine secretion in RAW264.7 cells. As illustrated in Figure 4C–F, LPS stimulation significantly upregulated the expression levels of the pro-inflammatory factors IL-18, IL-6, IL-1β, and TNF-α in RAW264.7 cells. However, treatment with TT-55 at a concentration of 60 μM significantly reduced the expression levels of IL-18, IL-6, IL-1β, and TNF-α in the cell culture supernatants of RAW264.7 cells. These results were consistent with previous testing. Upon co-treatment with the Nrf2 pathway inhibitor ML385, the inflammatory cytokine content in the RAW264.7 cell culture supernatant increased compared to the LPS + TT-55 group. This suggested that the anti-inflammatory effect of TT-55 might be mediated via activation of the Nrf2 pathway.

### 2.5. TT-55 Inhibits LPS-Induced NLRP3 Inflammasome in RAW264.7 Cells via Nrf2

**Figure 5:**
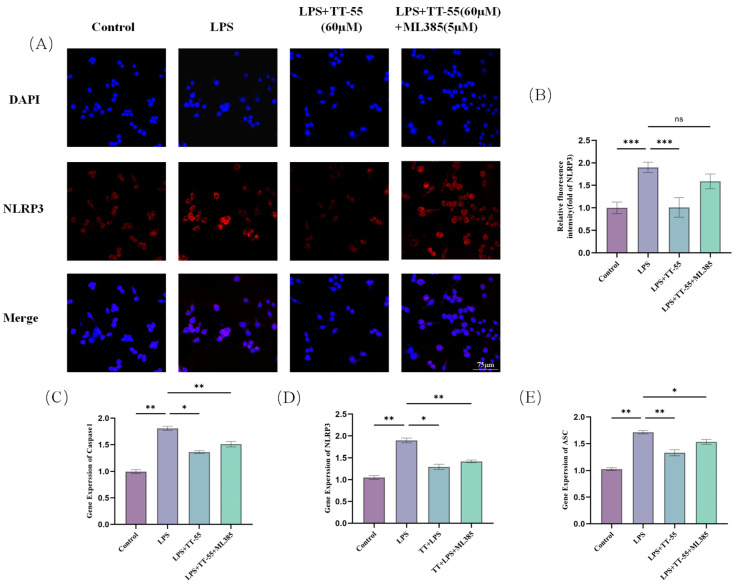
TT-55 inhibits LPS-induced NLRP3 inflammasome in RAW264.7 cells via Nrf2/HO-1. (**A**,**B**) Immunofluorescence analysis of NLRP3 expression. (**C**–**E**) mRNA expression levels of three inflammasome components (NLRP3, ASC, caspase-1) in RAW 264.7 cells were analyzed by qPCR under Nrf2 pathway inhibitor treatment. Each experiment was independently performed in triplicate at minimum, and all results are presented as mean ± standard deviation. * *p* < 0.05, ** *p* < 0.01, *** *p* < 0.001 compared with the LPS group.

The image obtained by laser confocal microscopy is shown in Figure 5A. TT-55 pretreatment suppressed LPS-induced NLRP3 inflammasome upregulated expression. In RAW264.7 cells pretreated with the Nrf2-specific inhibitor ML385, NLRP3 inflammasome expression increased, indicating that inhibiting Nrf2 expression promotes NLRP3 inflammasome expression. TT-55 also significantly downregulates the expression of inflammatory mediators, including pro-inflammatory cytokines, such as NO, IL-18, IL-6, and IL-1β, by modulating the Nrf2 signaling pathway in LPS-stimulated RAW264.7 cells. TT-55 significantly attenuated the LPS-induced upregulation of key genes associated with the NLRP3 inflammasome in RAW 264.7 macrophages. Our findings indicate that TT-55 downregulates the expression of NLRP3 inflammasome-related genes.

## 3. Discussion

Based on our prior research findings, an endophytic fungus of mangrove trees named ‘*Phomopsis asparagi* DHS-48’ exhibits immense potential for producing novel compounds. To further explore this fungus’s capacity for generating additional unknown secondary metabolites, we subjected it to epigenetic regulation. Phaseolorin J (TT-55), a chromone compound isolated and purified from the fermentation products of epigenetically regulated *Phomopsis asparagi*, exhibits significant inhibitory effects on the proliferation of ConA-induced T cells and LPS-induced B cells in mouse spleen lymphocytes while demonstrating low toxicity towards mouse splenic lymphocytes. Its structure was characterized by one-dimensional NMR spectroscopy and mass spectrometry (Figures S1–S4). This study employed LPS-induced RAW264.7 macrophages to establish an in vitro model of oxidative stress and inflammatory response. The preliminary findings indicate that LPS stimulates the secretion of inflammatory cytokines as well as the expression of inflammatory genes and proteins. TT-55 treatment significantly attenuated these effects. This finding is consistent with reports on other natural chromone compounds, such as astragalo side IV [36], glycyrrhizin [37], baicalein [38], and quercetin [39], which not only reduced LPS-induced NO production and pro-inflammatory cytokine production (TNF-α, IL-6, IL-1β) in RAW264.7 cells but also inhibited mRNA expression of pro-inflammatory cytokines. This aligns with our findings on TT-55’s anti-inflammatory activity. Excessive ROS production in cells and tissues disrupts the antioxidant system, leading to cellular damage. Concurrently, excessive ROS generation triggers inflammatory responses by promoting the synthesis and release of pro-inflammatory cytokines. In this process, LPS acted as an activator of ROS, regulating related oxidative stress factors to mediate inflammatory responses. Ioana demonstrated that curcumin reduced oxidative stress by scavenging ROS and enhancing antioxidant element expression [40]. Flow cytometry analysis revealed that LPS promotes substantial ROS release in RAW264.7 cells, an effect inhibited by TT-55. This discovery is consistent with the research results on the anti-inflammatory and antioxidant effects of curcumin. Mohammad demonstrated that galangin prevents oxidative stress responses by reducing malondialdehyde and nitric oxide levels while also decreasing inflammatory mediators in cells [41]. Following TT-55 pretreatment, MDA expression decreased while SOD and HO-1 activity increased. This demonstrated TT-55’s potent anti-inflammatory and antioxidant functions in cellular inflammatory responses and oxidative stress. However, its molecular mechanisms remain complex and warrant further investigation.

**Figure 6:**
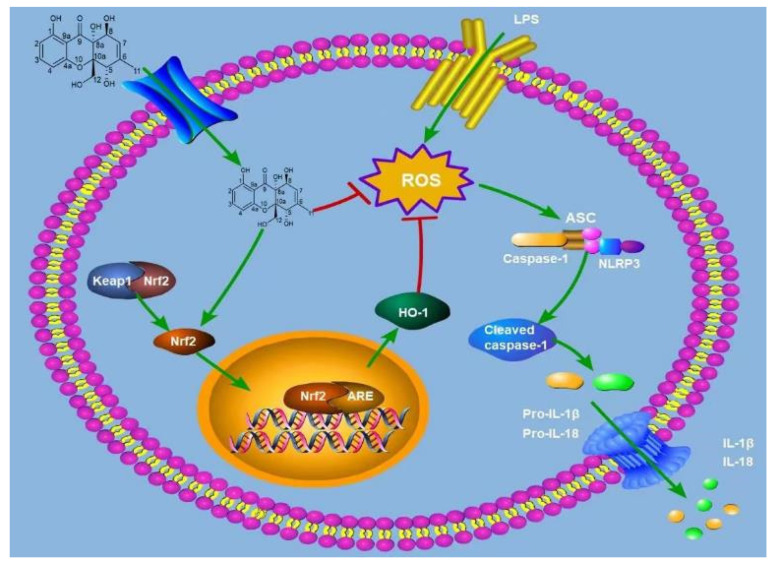
Schematic diagram of the mechanism by which TT-55 suppresses NLRP3 inflammasome expression and alleviates inflammatory and oxidative responses through activation of the Nrf2 signaling pathway.

As a redox-sensitive basic leucine zipper transcription factor, Nrf2 plays a central regulatory role in cellular protective mechanisms. This factor not only effectively suppresses oxidative stress responses but also exhibits significant anti-inflammatory effects. Simultaneously, as a key regulatory element, it profoundly influences the expression of multiple cell-protective genes. Under static conditions, Nrf2 interacts with Keap1 and remains in an inactive state [42]. During oxidative stress and inflammatory responses, Nrf2 dissociates from the Keap1 complex and is subsequently transported into the cell nucleus. Once nuclearized, Nrf2 exerts its biological functions by regulating the transcriptional activity of multiple cell protective genes, including the upregulation of key antioxidant enzymes, such as HO-1 and NQO1, thereby effectively counteracting oxidative damage and inflammatory reactions. HO-1 prevents inflammatory escalation by degrading heme groups into products like bilirubin, which exerts protective antioxidant effects [43]. As a pigment protein widely distributed across diverse cells, heme plays a vital role in cellular metabolism. Studies reveal that activating the Nrf2 signaling pathway and upregulating HO-1 effectively inhibits NLRP3 inflammasome activation. Significant increases in NLRP3, ASC, and caspase-1 mRNA expression levels were observed in LPS-stimulated RAW264.7 cells, confirming that LPS-induced oxidative stress promotes inflammasome upregulation. After receiving TT-55 treatment, the expression significantly decreased. This indicates that TT-55 not only exerts antioxidant effects but also suppresses the expression of NLRP3 inflammasome-related genes. ML385 acts as an inhibitor of Nrf2 in various cells and tissues [44]. We further investigated whether TT-55 modulates LPS-induced inflammation and oxidative stress in RAW264.7 cells via the Nrf2 signaling pathway using ML385. The reduction in the antioxidant SOD and the increase in MDA directly reflect cellular oxidative stress levels. In our study, we observed that TT-55 significantly increased SOD levels and decreased MDA levels in RAW264.7 cells. However, these effects were suppressed by the Nrf2 inhibitor ML385. Therefore, we speculate that TT-55 may exert its protective effects by reducing Nrf2-associated oxidative stress. Liu X N found that isoliquiritinide treatment increased Nrf2 and HO-1 expression levels, promoted glutathione and superoxide dismutase production, and improved histopathological manifestations in pancreatic tissues of male ICR mice with acute pancreatitis. However, ML385 abolished the effects of isoliquiritin on pancreatitis. Inhibition of Nrf2 attenuated the protective effects of isoliquiritin [45]. This aligns with our findings. Concurrently, ML385 significantly reversed the TT-55-inhibited expression of LPS-induced inflammatory factors in RAW264.7 cells. Following LPS stimulation in RAW264.7 macrophages, we observed significant elevations in the levels of inflammatory mediators (IL-1β, TNF-α, IL-18, IL-6) and increased mRNA expression of genes associated with the NLRP3 inflammasome pathway. TT-55 treatment significantly elevated the levels of Nrf2 and HO-1, promoted the nuclear translocation of Nrf2, and suppressed both the upregulation of NLRP3 inflammasome-related genes and the secretion of pro-inflammatory cytokines, including IL-1β, TNF-α, IL-18, and IL-6. It facilitates the nuclear translocation of Nrf2 via activation of the Nrf2/HO-1 signaling pathway, which in turn suppresses the expression of NLRP3 inflammasome-associated genes and pro-inflammatory cytokines such as IL-1β, TNF-α, IL-18, and IL-6. Nrf2 is an effective transcription activator that regulates the transcription of numerous antioxidant genes in response to electrophilic and oxidative stress. TT-55 treatment enhances Nrf2 nuclear translocation and increases cellular expression of HO-1 and Nrf2. However, the Nrf2 inhibitor ML385 significantly blocked TT-55’s protective effects against inflammatory responses and oxidative stress in RAW264.7 cells. These findings further demonstrate that TT-55 suppresses cellular inflammation and oxidative stress by activating the Nrf2 pathway. As illustrated in Figure 6, it is worth noting that in LPS-stimulated RAW264.7 macrophages, TT-55 demonstrated efficacy comparable to that of two classic and widely used antioxidants and anti-inflammatory agents, N-acetylcysteine (NAC) and resveratrol, in reducing intracellular oxidative stress and exerting anti-inflammatory effects [46,47]. In addition, given that RAW264.7 cells were stimulated with LPS before TT-55 treatment, it is reasonable to infer that TT-55 does not merely block LPS membrane receptors but may act at the post-receptor signaling level to inhibit LPS-induced inflammatory and cytotoxic responses. Further studies are warranted to identify whether TT-55 directly interacts with Keap1, interferes with upstream signaling mediators, or affects other receptor-dependent pathways involved in LPS action. It is worth noting that TT-55 may possess the structural characteristics typical of PAINS (pan-interfering compounds); compounds with such structural features interact with multiple intracellular targets in a dose-dependent manner. The conclusion that TT-55 exerts a specific effect solely on the antioxidant and anti-inflammatory system (without affecting other pathways) has not yet been confirmed. It can, therefore, be inferred that TT-55 may possess multi-target activity.

## 4. Materials and Methods

### 4.1. Materials

Specific optical rotation was determined using an ATR-W2 HHW5 digital Abbe refractometer (Shanghai Physical Optics Instrument Factory, Shanghai, China). Ultraviolet spectra were measured with a Shimadzu UV-2600 PC spectrophotometer (Shimadzu Corporation, Tokyo, Japan), while ECD spectra were obtained on a JASCO J-715 spectrophotometer (JASCO, Tokyo, Japan). ^1^H, ^13^C, and ^2^D NMR spectra were recorded on a Bruker AV 400 NMR spectrometer using TMS as the internal standard. High-resolution electrospray ionization–mass spectrometry (ESI-MS) was performed on a Shimadzu LCMS-IT-TOF instrument (Tokyo, Japan) using peak matching. Thin-layer chromatography (TLC) was performed using silica gel (200–400 mesh, Qingdao Ocean Chemical Co., Ltd., Qingdao, China). Column chromatography (CC) was performed using Sephadex LH-20 (18–110 μm, Merck KGaA, Darmstadt, Germany). High-performance liquid chromatography (HPLC) was conducted using a Waters e2695 system (Waters Corporation, Milford, MA, USA).

The Laser Confocal Microscope TCS-SP5 (Leica, Germany), fully automatic inverted differential interference microscope DMI8 (Leica, Germany), Flow Cytometer Novocyte Quanteon VBYR (Agilent, Singapore), Real-time PCR System CFX Connect (Bio-Rad, Hercules, CA, USA), Gel Imaging System (Protein Analysis) Amersham Imager (GE Healthcare, Chicago, IL, USA), and Microplate Reader KHB-ST-360 (Kehua, Shanghai, China) were used. RAW264.7 cells, high-quality fetal bovine serum, high-glucose DMEM medium, penicillin/streptomycin, and phosphate-buffered saline (PBS) were purchased from Wuhan Procell Life Technology Co., Ltd. (Wuhan, China). ELISA kits for nitric oxide (NO), malondialdehyde (MDA), superoxide dismutase (SOD), and reactive oxygen species (ROS), along with other experimental reagents, including lipopolysaccharide (LPS), phenylmethanesulfonylfluoride (PMSF), enhanced RIPA lysis buffer, dimethyl sulfoxide (DMSO), DAPI staining solution, SDS-PAGE gel preparation kit, SDS-PAGE rapid running buffer, Western blot rapid transfer buffer, blocking buffer, TBST buffer, and primary/secondary antibody diluents, were purchased from Beyotime Biotechnology Co., Ltd. (Shanghai, China). ELISA kits for interleukin-1β (IL-1β), interleukin-18 (IL-18), interleukin-6 (IL-6), and tumor necrosis factor-α (TNF-α) were purchased from Fusheng Industrial Co., Ltd. (Shanghai, China). ELISA kits targeting cyclooxygenase-2 (COX-2), inducible nitric oxide synthase (INOS), and heme oxygenase-1 (HO-1) were purchased from Wuhan Elabscience Biotechnology Co., Ltd. (Wuhan, China). ML385 was obtained from Sigma-Aldrich (St. Louis, MO, USA). The Cell Counting Kit-8 (CCK-8) was purchased from White Shark Biosharp Co., Ltd. (Shanghai, China). Primary antibodies against Nrf2 and NLRP3, as well as horseradish peroxidase (HRP)-conjugated anti-rabbit IgG secondary antibody, were procured from Wuhan Proteintech Biotechnology Co., Ltd. (Wuhan, China).

### 4.2. Fungal Material

The endophytic fungus *Phomopsis asparagi* DHS-48 was isolated from fresh hypocotyls of the mangrove plant Rhizophora mangle, collected from the Dongzhai Harbor Mangrove Nature Reserve in Hainan, China. The strain (Voucher No. DHS-48) was identified using molecular biology methods, specifically through DNA amplification and sequencing of the ITS region. The resulting sequence has been deposited in GenBank under accession number MT126606. The endophytic fungus Pestalotiopsis sp. HHL-101 was isolated from fresh branches of the mangrove plant Rhizophora stylosa, also collected from the Dongzhai Harbor Mangrove Nature Reserve. This strain (Voucher No. HHL-101) was similarly identified via DNA amplification and ITS sequencing, with the sequence deposited in GenBank under accession number EF451799.

### 4.3. Fermentation, Extraction, and Isolation

Single colonies of *Phomopsis asparagi* DHS-48 and Pestalotiopsis sp. HHL-101 was initially inoculated separately onto potato dextrose agar (PDA) plates and cultured at 28 °C for 5 days. Subsequently, agar plugs (approx. 1 cm^2^) containing mycelia from both DHS-48 and HHL-101 were inoculated into 1 L Erlenmeyer flasks (160 flasks total). Each flask contained a sterilized solid medium composed of rice (100 g), peptone (0.6 g), crude sea salt (3 g), and water (100 mL). The co-culture fermentation was carried out at 28 °C for 30 days. The fermented cultures from all 160 flasks were extracted three times with EtOAc (400 mL per flask). The organic extracts were combined and evaporated under reduced pressure to yield a crude extract (90 g). To remove lipophilic impurities, the crude extract was suspended in 1 L of 90% MeOH/H_2_O and partitioned three times with an equal volume of petroleum ether (PE). The PE layer was discarded, and the aqueous methanol layer was concentrated under reduced pressure to afford a defatted extract (70 g). The defatted extract was mixed with silica gel (100–200 mesh) at a ratio of 1.5:1 (silica/extract, *w*/*w*) and subjected to silica gel column chromatography (CC). The column was eluted with a CH_2_Cl_2_–MeOH gradient system (30:1, 25:1, 20:1, 15:1, 10:1, 8:1, 6:1, 3:1, and 1:1, *v*/*v*). Each gradient step utilized 4 L of solvent, and fractions were collected in 1 L increments. After concentration, 36 sub-fractions were obtained. Based on TLC analysis, fractions with similar profiles were combined to give five main fractions (Fr. 1–Fr. 5). Fraction 5 was further fractionated via silica gel CC eluting with CH_2_Cl_2_–EtOAc (1:1, *v*/*v*) to yield six sub-fractions (Fr. 5.1–Fr. 5.6). Fr. 5.3 was purified using Sephadex LH-20 CC (CH_2_Cl_2_–MeOH, 1:1, v/v), followed by semi-preparative RP-HPLC (MeOH–H_2_O, 40:60, *v*/*v*) to yield compound Phaseolorin J (20 mg).

### 4.4. Identification of TT-55

Phaseolorin J (TT-55): light yellow amorphous powder; [α]^20^_D_ +160 (c 0.01, MeOH); UV (MeOH) λmax 214 nm. ^1^H NMR (400 MHz, CD_3_OD) δ 7.38 (t, *J* = 8.2 Hz, 1H, H-3), 6.52 (d, *J* = 8.2 Hz, 1H, H-4), 6.43 (d, *J* = 8.2 Hz, 1H, H-2), 5.61 (dq, *J* = 4.8, 1.7 Hz, 1H, H-7), 4.69 (br s, 1H, H-5), 4.55 (d, *J* = 4.8 Hz, 1H, H-8), 4.12 (d, *J* = 13.2 Hz, 1H, H-12a), 4.03 (d, *J* = 13.2 Hz, 1H, H-12b), 1.86 (d, *J* = 1.7 Hz, 3H, H-11); ^13^C NMR (100 MHz, CD_3_OD) δ 197.2 (C-9), 163.2 (C-1), 160.8 (C-4a), 140.5 (C-6), 138.9 (C-3), 122.3 (C-7), 109.3 (C-2), 108.7 (C-4), 108.4 (C-9a), 86.5 (C-10a), 74.5 (C-8a), 74.3 (C-5), 67.9 (C-8), 64.1 (C-12), 19.2 (C-11). HRESIMS m/z 331.0780 [M + Na]^+^ (calcd for C_15_H_16_O_7_Na 331.0788). The chemical structure of TT-55 is shown in Figure 1A.

### 4.5. Cell Culture and Treatment

RAW264.7 cells were cultured in high-glucose Dulbecco’s Modified Eagle Medium (DMEM) supplemented with 10% fetal bovine serum (FBS), 100 U/mL penicillin, and 100 μg/mL streptomycin. The cells were then maintained in a humidified incubator at 37 °C with 5% CO_2_. Before experimentation, TT-55 was prepared as a one mM stock solution in DMSO, subsequently diluted with PBS to achieve experimental concentrations of 20 μM, 40 μM, and 60 μM. All experiments employed LPS stimulation at a concentration of 100 ng/mL.

### 4.6. CCK-8 Assay

When the RAW264.7 cell density reached 80% confluence in the T25 cell culture flask, the cells were harvested via pipetting and centrifugation to prepare a cell suspension. The cells are counted and diluted to a density of 1 × 10^5^ cells/mL before being seeded into a 96- well plate. Each well receives 200 μL of medium; the blank control group receives 200 μL of cell suspension. The drug control group received 200 μL medium and TT-55 (without cells); the drug group contained 200 μL of cell suspension diluted with different concentrations of TT-55 (with cells). After culturing until cells were fully adhered, TT-55 was added and incubated for 24 h. Subsequently, 20 μL of CCK-8 reagent was added to each well, incubated in a cell culture incubator for 2 h, and then removed and placed in an enzyme-linked immunosorbent assay (ELISA) reader to measure the absorbance at 450 nm.

### 4.7. NO Assay

Perform cell counting and dilute the cell suspension to a density of 1 × 10^5^ cells/mL. Seed cells into pretreated 96-well plates at 200 μL per well. Once the cells have fully adhered, treat them with LPS for 6 h, followed by treatment with TT-55 for 2 h. Subsequently, collect the supernatant from each well and mix with Griess reagent. Incubate at room temperature for 3–5 min. Measure the absorbance at 540 nm using a microplate reader.

### 4.8. Assessment of Oxidative Stress

Count RAW264.7 cells and dilute the cell suspension to a density of 1 × 10^6^ cells/mL. Seed cells into a 6-well plate and incubate. Six hours after stimulating the cells with LPS, add TT-55 and treat for 2 h. Then, detach the cells, add 500 μL DCFH-DA, and incubate in a cell culture incubator at 37 °C for 20 min. Wash cells three times with serum-free cell culture medium to thoroughly remove unincorporated DCFH-DA. Detect fluorescence intensity using a flow cytometer with the FITC detection channel set to an excitation wavelength of 488 nm and an emission wavelength of 525 nm. Analyze flow cytometry results using FlowJo software. Antioxidant malondialdehyde (MDA) and superoxide dismutase (SOD) production were measured using chemical assay kits.

### 4.9. Enzyme-Linked Immunosorbent Assay (ELISA)

After LPS, to stimulate cells for 6 h, add 2 h of TT-55 treatment. Collect the cell culture supernatant from all RAW 264.7 cell groups in 6-well plates. Detect levels of inflammatory cytokines TNF-α, IL-1β, IL-18, and IL-6 using the kit.

### 4.10. qPCR Analysis

RAW 264.7 cells were seeded into 6-well plates, stimulated with LPS for 6 h, and then treated with various concentrations of TT-55 for an additional 2 h. Total RNA was extracted from RAW 264.7 cells using the Rapid Animal Tissue/Total RNA and was isolated from RAW264.7 cells using the Rapid Animal Tissue/Cell Total RNA Extraction Kit (Beyotime, Shanghai, China). Reverse transcription was conducted using a kit supplied by Sevier (Wuhan, China), followed by polymerase chain reaction (PCR) amplification. The reaction was denatured at 95 °C for 30 s, followed by 40 consecutive thermal cycles (95 °C for 15 s; 60 °C for 30 s). Primers were:
β-actinF:CGGTTCCGATGCCCTGAGGCTCTTR:CGTCACACTTCATGATGGAATTGAIL-1βF:GTTGACGGACCCCAAAAGATR:CCTCATCCTGGAAGGTCCACIL-6F:GGTGACAACCACGGCCTTCCCR:AAGCCTCCGACTTGTGAAGTGGTTNF-αF:TATGGCTCAGGGTCCAACTCR:CTCCCTTTGCAGAACTCAGGNrf2F:AGCAGGACATGGAGCAAGTTR:TTCTTTTTCCAGCGAGGAGANLRP3F:TTCTGCCGTGGTCTCTTCTCAR:TGCCTCACACAGCACCCTCCaspase-1F:CTGGGACCCTCAAGTTTTGCR:AGACGTGTACGAGTGGTTGTASCF:TTGCTGGATGCTCTGTATGGR:CCAAGTAGGGCTGTGTTTGCIL-18F:CCTGACATCTTCTGCAAAGGR:GCTGTCTTTTGTCAACGAACA

### 4.11. Immunofluorescence

Seed RAW264.7 cells at a density of 10^5^ cells/mL in laser confocal microplates and incubate overnight for 24 h to allow cell attachment. After LPS, to stimulate cells for 6 h, add 2 h of TT-55 treatment, followed by three consecutive 5 min washes with PBS. Subsequently, the cells were fixed with 4% paraformaldehyde solution for 25 min at room temperature. Following fixation, cells were washed three times with PBS, each wash lasting 5 min. Finally, cells were treated with 0.1% Triton X-100 for 10 min, followed by three 5 min washes with PBS. After blocking with immunofluorescence blocking solution for 10 min, primary antibodies (anti-Nrf2 and anti-NLRP3) were incubated overnight. On the following day, after being washed three times with PBS (5 min per wash), the samples were incubated with secondary antibodies (anti-Nrf2 and anti-NLRP3) for 60 min in the dark. Wash three times with PBS for 5 min each. Stain with DAPI at room temperature in the dark for 5 min. Wash three times with PBS for 5 min each. NLRP3 immunofluorescence images were captured using a Laser Confocal Microscope TCS SP5 (Leica, Germany). Nrf2 immunofluorescence images were captured using a fully automatic inverted differential interference contrast microscope (Leica DMI8). Quantitative analysis of fluorescence intensity was performed using Fiji (ImageJ 1.54f, National Institutes of Health, Bethesda, MD, USA) software. For each group, the mean fluorescence intensity (MFI) was first measured. After subtracting background fluorescence, the signal intensity was normalized by the number of cells in the corresponding field of view. Relative fluorescence intensity was calculated by comparing the results with those of the control group.

### 4.12. Western Blot Analysis

RAW 264.7 cells were seeded in 6-well plates. After 6 h of LPS stimulation, the cells were treated with different concentrations of TT-55 for 2 h. Extract nuclear proteins using the Nuclear and Cytoplasmic Protein Extraction Kit (Beyotime, Shanghai, China). Perform Western blotting using an anti-Nrf2 antibody. Perform protein quantitative analysis using ImageJ software.

### 4.13. Statistical Analysis

Each experiment was independently replicated at least three times to ensure reproducibility. Data visualization was performed using GraphPad Prism 9, and one-way analysis of variance (ANOVA) was applied to assess significant differences among groups. All results are presented as mean ± standard deviation. Statistical significance was set at *p* < 0.05.

## 5. Conclusions

This study first identified TT-55, a chromone compound derived from the secondary metabolites of mangrove endophytic fungi, which exhibits inhibitory effects on LPS-induced inflammatory responses in RAW264.7 cells with low toxicity. It also suppresses LPS-induced oxidative stress in RAW264.7 cells. This study is the first to demonstrate that TT-55 promotes the nuclear translocation of Nrf2 via the Nrf2/HO-1 signaling pathway, thereby upregulating the expression of its downstream antioxidant effector HO-1 and ultimately suppressing the upregulation of NLRP3 inflammasome-related genes, thus exerting both antioxidant and anti-inflammatory effects. However, since all the experiments were conducted only in RAW264.7 macrophages, further in vivo studies are needed to verify the anti-inflammatory and antioxidant activities of TT-55.

## Data Availability

The original data presented in the study are included in the article/Supplementary Materials; further inquiries can be directed to the corresponding author.

## Supplementary Materials

The following supporting information can be downloaded at: https://www.mdpi.com/article/10.3390/md24040130/s1, Figure S1, HPLC chromatography of phaseolorin J; Figure S2, 1H-NMR of phaseolorin J; Figure S3, 13C-NMR of phaseolorin J; Figure S4, HR-ESI-MS of phaseolorin J; Figure S5, prestained protein marker (8–200 kDa); Figure S6, original protein image of LabminB (66 kDa) detected using the Amersham 600 QC imager; Figure S7, original protein image of Nuc-Nrf2 (110 kDa) detected using the Amersham 600 QC imager.
