# Supplementary material for: Phaseolorin J Alleviates Cellular Inflammation and Oxidative Stress by Inhibiting NLRP3 Inflammasome Expression via the Nrf2/HO-1 Pathway
Source: Mar Drugs. 2026 Mar 31;24(4):130. doi: 10.3390/md24040130 (PMC13118055; doi:10.3390/md24040130)
Supplement: Supplementary file 1 [file marinedrugs-24-00130-s001.zip › marinedrugs-4194960-supplementary.pdf]

Figure S1. HPLC chromatography of phaseolorin J (**1**)--- 4
Figure S2. ^{1}H-NMR of phaseolorin J (**1**) --- 4
Figure S3. ^{13}C-NMR of phaseolorin J (**1**)-- 4
Figure S4. HR-ESI-MS of phaseolorin J (**1**)-- 7

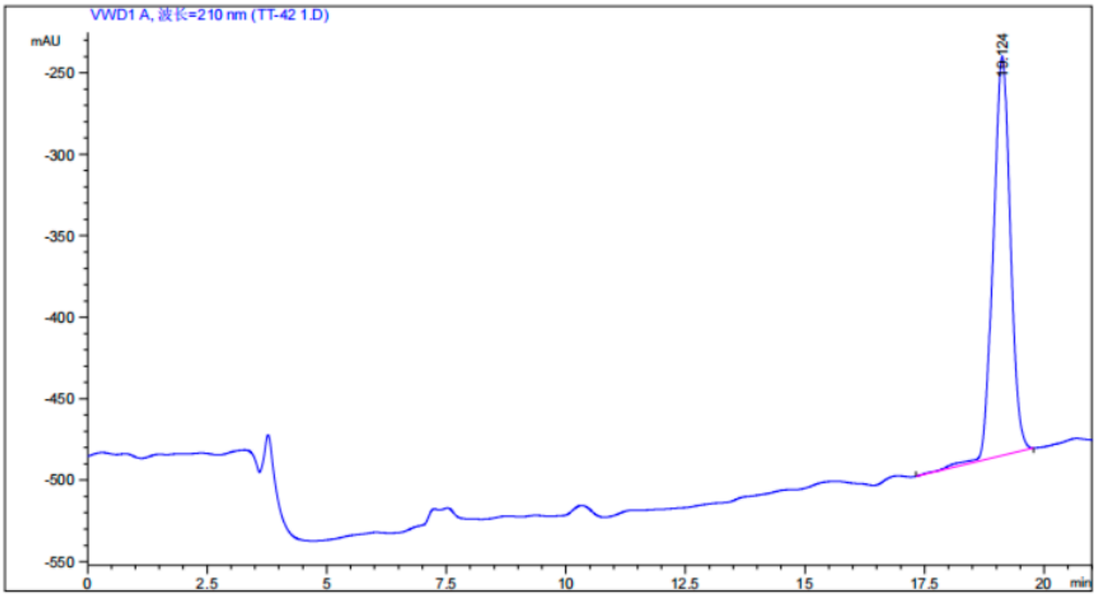

Peak	Retention time	Peak type	Peak width	Peak area	Peak height	Peak area%
#	[min]		[min]	mAU *s	[mAU]	%
1	19.124	BB	0.3732	6126.05273	245.00531	100.0000

Figure S1. HPLC chromatography of phaseolorin J (**1**)

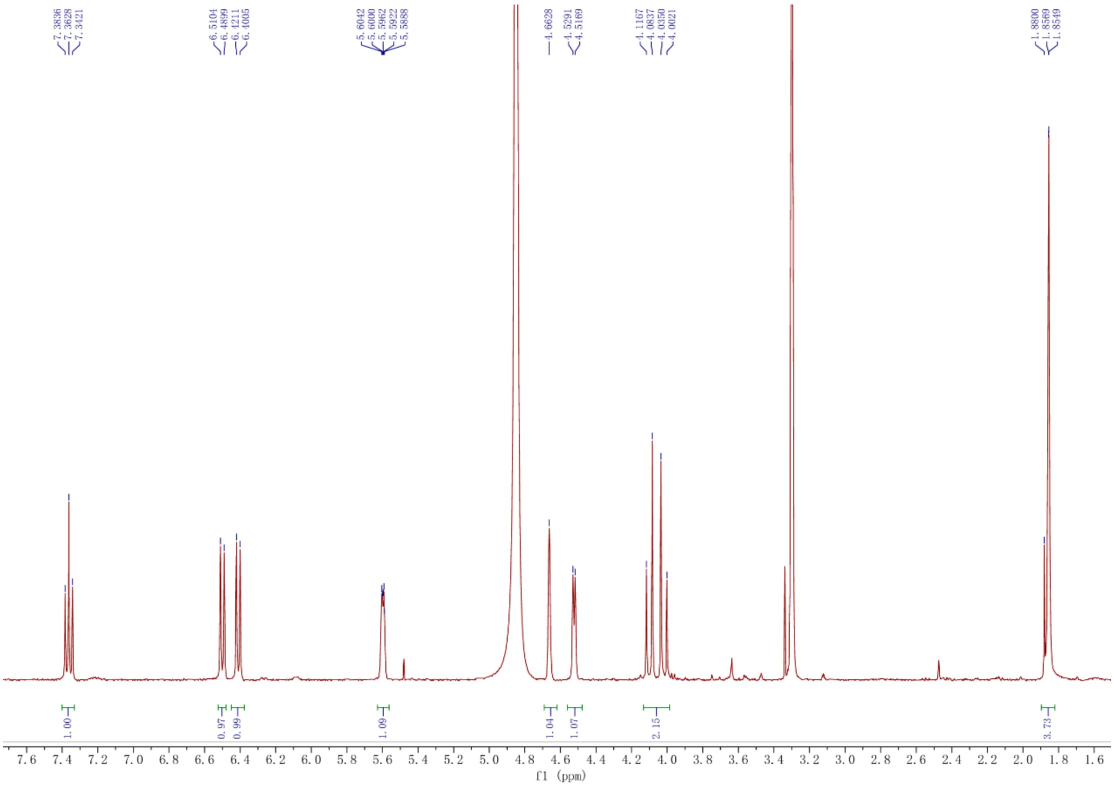

Figure S2. ^{1}H-NMR of phaseolorin J (**1**)

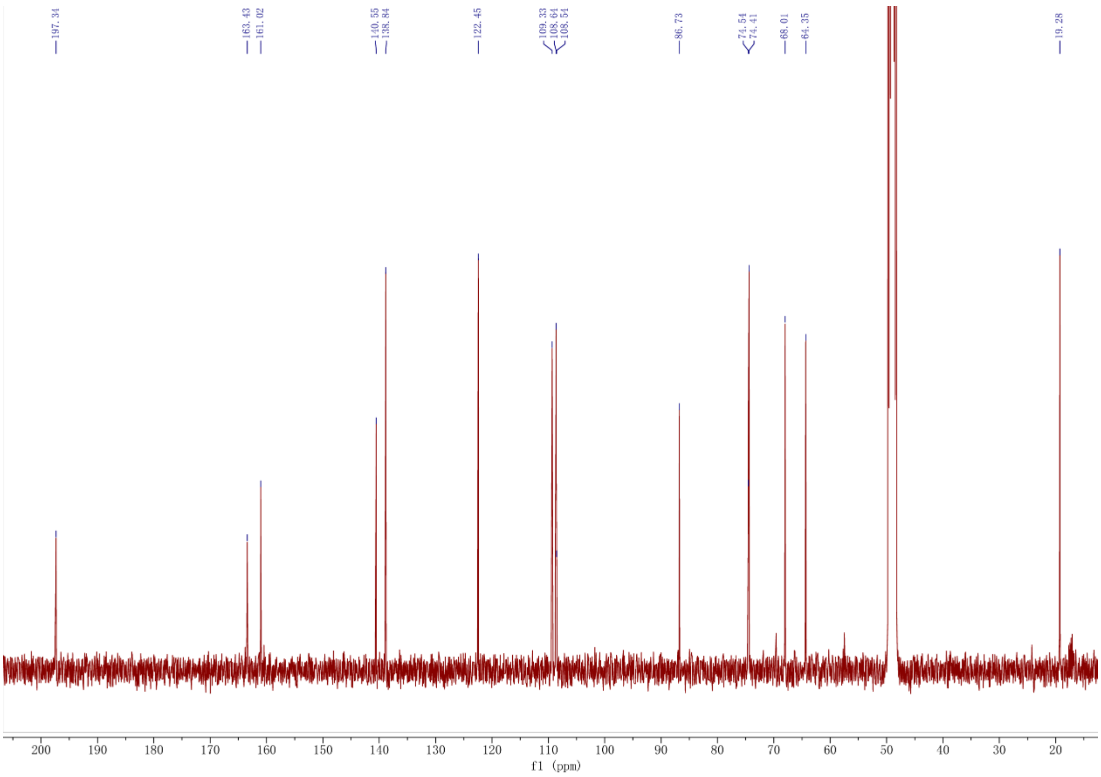

Figure S3. ^{13}C-NMR of phaseolorin J (**1**)

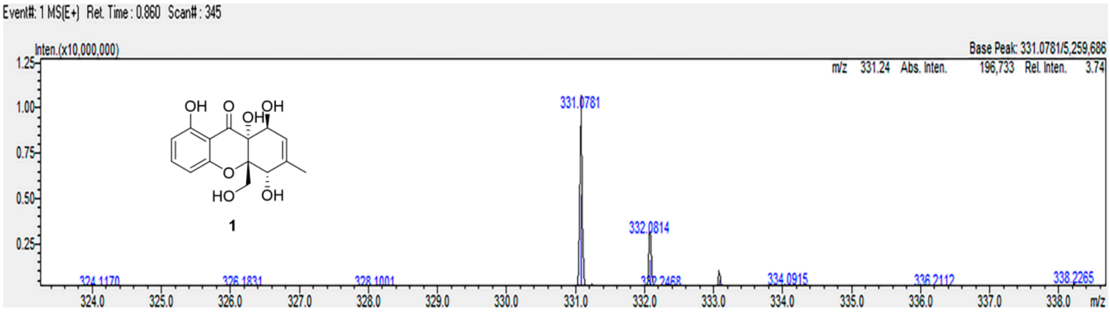

Figure S4. HR-ESI-MS of phaseolorin J (**1**)
